# Carbon source diversity shapes bacterial interspecies interactions

**DOI:** 10.1093/ismejo/wraf224

**Published:** 2025-10-08

**Authors:** Hiroki Ono, Saburo Tsuru, Chikara Furusawa

**Affiliations:** Department of Biological Sciences, Graduate School of Science, The University of Tokyo, 7-3-1 Hongo, Bunkyo-ku, Tokyo 113-0033, Japan; Universal Biology Institute, Graduate School of Science, The University of Tokyo, 7-3-1 Hongo, Bunkyo-ku, Tokyo 113-0033, Japan; Universal Biology Institute, Graduate School of Science, The University of Tokyo, 7-3-1 Hongo, Bunkyo-ku, Tokyo 113-0033, Japan; Center for Biosystems Dynamics Research, RIKEN, 6-7-1 Minatojima-minamimachi, Chuo-ku, Kobe 650-0047, Japan

**Keywords:** bacterial community, synthetic community, interspecies interaction, carbon source diversity, resource competition

## Abstract

Bacterial communities exhibit various classes of interspecies interactions, ranging from synergistic to competitive. As these interaction classes play a crucial role in determining characteristics of bacterial communities, including species composition and community stability, understanding the mechanisms that shape them is important. Whereas several studies have suggested that synergistic interactions are rare, a study focused on single-carbon-source environments reported them to be relatively common. This discrepancy highlights the potential role of carbon source diversity in shaping interaction classes, although the quantitative relationship remains unclear. To elucidate this relationship, we examined 896 interspecies interactions amongst 28 synthetic bacterial pairs, isolated from various environments, under 32 conditions with varying levels of carbon source diversity. As a result, we frequently observed synergistic interactions in single-carbon-source environments, with the interactions shifting to competitive as the carbon source diversity increased. Further analyses suggested that this shift was driven by processes occurring in environments with an increased diversity of carbon sources, such as resource competition. Our findings provide new insights into how environmental factors, particularly carbon source diversity, shape interspecies interactions in bacterial communities.

## Introduction

Many bacteria coexist with multiple species, forming complex communities where they interact either indirectly through their growth environment [1, 2] or directly via physical contact [3, 4]. These interspecies interactions include various classes [5–7], ranging from competition, in which both species inhibit growth of each other, to mutualism, in which they promote growth for both species. The interaction classes affect species composition and community stability [8–14], which in turn plays a key role in essential functions of natural communities, such as global nutrient cycling [15] and food digestion [16], as well as applications of synthetic communities, including bioremediation [17] and bacteriotherapy [18]. Therefore, it is crucial to quantitatively understand how different interaction classes emerge under specific conditions for the effective utilisation of both natural and synthetic communities [19, 20].

To understand the principles governing interaction classes, previous studies primarily focused on combinations of bacterial pairs and growth environments. Several studies simulated bacterial interactions under various growth environments, suggesting that synergistic interactions—such as mutualism (bidirectional growth facilitation) and commensalism (unidirectional growth facilitation)—can form to some extent via their metabolites produced in response to the growth environments [21–24]. Furthermore, the Black Queen Hypothesis [25]—which proposes that some bacteria lose costly functions via gene lose and instead depend on others—suggests that synergistic interactions mediated by such metabolites may be ubiquitous in bacterial communities.

Many studies that experimentally assessed bacterial interactions, however, reported that synergistic interactions were rare [26–30]. In these studies, bacterial interactions were examined using synthetic pairs of two isolated species. Each of these studies examined a single natural medium containing multiple carbon sources and reported that competitive interactions—such as competition (bidirectional growth inhibition) and amensalism (unidirectional growth inhibition)—occurred predominantly. These results made it seem that synergistic interactions via secreted metabolites, which were theoretically predicted to occur frequently, were rare [31].

A previous study investigated synthetic bacterial pairs under well-defined media containing only a single carbon source selected from 33 different organic compounds [32]. The study reported that synergistic interactions were more common in single-carbon-source environments, contrasting with studies conducted in multi-carbon-source environments [26–30]. In that work, exploitation (parasitism)—consisting of unidirectional growth facilitation and unidirectional growth inhibition—was classified as a positive interaction, which differs from our classification scheme. These findings led us to hypothesise that bacterial interaction classes are shaped by the carbon source diversity. Specifically, synergistic interactions are more likely to occur in environments with low carbon source diversity [32], whereas competitive interactions become more prevalent as the carbon source diversity increases [26–31]. However, few studies have systematically examined bacterial interactions across different environments, particularly those with varying carbon source diversity while controlling for other confounding environmental factors. Therefore, the quantitative relationship between bacterial interactions and carbon source diversity remains largely unexplored.

**Figure 1:**
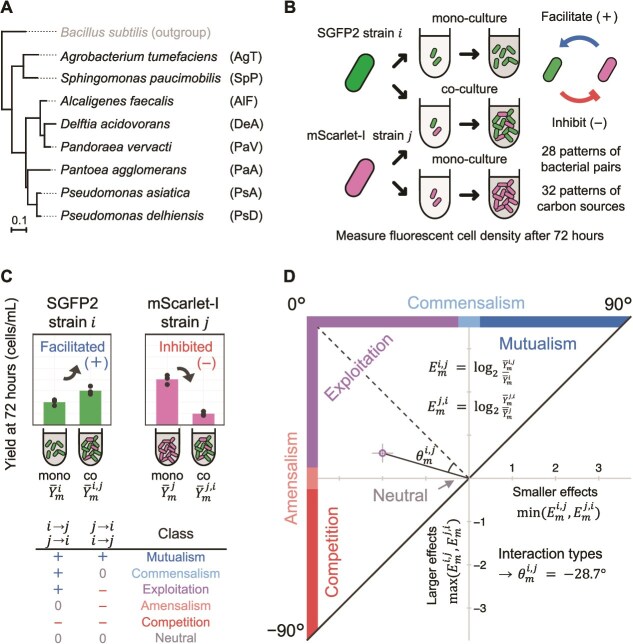
Overview of methods for measuring and analysing interspecies interactions. (A) Phylogenetic tree of the eight bacterial species used in this study, constructed based on 16S rRNA gene sequences. Abbreviations of species names used in this manuscript are shown on the right. The scale bar for this phylogenetic tree is shown in the bottom left of the panel. (B) Experimental setup for examining interspecies interactions. Mono-cultures (single-species) and co-cultures (two-species) were performed for each bacterial pair (species ${i}$ and ${j}$) in the growth environment ${m}$. Two unidirectional effects (from species ${i}$ to ${j}$ and from species ${j}$ to ${i}$) were evaluated by comparing the fluorescent cell densities in mono-cultures and co-cultures after 72 h of incubation. (C) Classification of unidirectional effects and bidirectional interactions. Fluorescent cell densities (${\overline{{Y}}}_{{m}}^{{i}}$ and ${\overline{{Y}}}_{{m}}^{{j}}$ for mono-cultures; ${\overline{{Y}}}_{{m}}^{{i},{j}}$ and ${\overline{{Y}}}_{{m}}^{{j},{i}}$ for co-cultures) were used to evaluate unidirectional effects by comparing the growth yields: facilitated (+), unaffected (0), or inhibited (−). Based on the combination of the unidirectional effects, bidirectional interactions were categorised into six classes. (D) Quantification of interspecies interactions. The unidirectional effects were quantified as the ${\log}_2$ ratios of co-culture yields to mono-culture yields, denoted as ${{E}}_{{m}}^{{i},{j}}$ and ${{E}}_{{m}}^{{j},{i}}$. On a Cartesian plane, the smaller effects ${\min}\left({{E}}_{{m}}^{{i},{j}},{{E}}_{{m}}^{{j},{i}}\right)$ are plotted on the horizontal axis, whereas the larger effects ${\max}\left({{E}}_{{m}}^{{i},{j}},{{E}}_{{m}}^{{j},{i}}\right)$ are plotted on the vertical axis. The interaction types ${{\theta}}_{{m}}^{{i},{j}}$ represent the angle that the line segment connecting this plotted point and the origin makes with the line where ${\max}\left({{E}}_{{m}}^{{i},{j}},{{E}}_{{m}}^{{j},{i}}\right)=-{\min}\left({{E}}_{{m}}^{{i},{j}},{{E}}_{{m}}^{{j},{i}}\right)$. Error bars represent the standard deviation across biological triplicates.

To understand how carbon source diversity affects bacterial interaction classes, we analysed 896 interactions amongst 28 bacterial pairs, composed of eight species isolated from diverse habitats under 32 different environments with varying carbon source diversity (Fig. 1A and B). Our results indicated that a broad range of interaction classes, from synergistic to competitive, were observed in single-carbon-source environments, whereas interactions shifted to competitive as carbon source diversity increased. Furthermore, detailed analyses suggested that these shifts were driven by intensified resource competition [33–36] in more complex media. This study offers new insights into the environmental determinants of bacterial interactions, especially the role of carbon source diversity. Additionally, our findings suggest that bacterial interactions can be manipulated through environmental modifications focusing on carbon source diversity, providing strategies for managing bacterial communities in biotechnological applications [37, 38].

## Materials and methods

### Culture media

All supplier information is provided in supplementary data (Table S1). All solutions were dissolved in pure water unless otherwise noted (Supplementary methods S1). To control the number of carbon sources in the growing environment, we used M9-based media prepared just before use. The media were prepared by dissolving the following components in 1.0 L of pure water: 100 ml of 10$\times$M9 salts solution (Na_2_HPO_4_ 67.78 mg/ml, KH_2_PO_4_ 30.00 mg/ml, NH_4_Cl 10.00 mg/ml, and NaCl 5.00 mg/ml, adjusted to pH 7.2 with 8.0 M NaOH), 1.0 ml of 1000$\times$Trace elements solutions (H_3_BO_3_ 24.8 μg/ml, MnCl_2_ 16.0 μg/ml, CoCl_2_ 7.2 μg/ml, (NH_4_)_6_Mo_7_O_24_ 3.4 μg/ml, ZnSO_4_ 2.8 μg/ml, and CuSO_4_ 2.4 μg/ml), 1.0 ml of MgSO_4_ solution (240.70 mg/ml), 1.0 ml of CaCl_2_ solution (11.10 mg/ml), and 1.0 ml of FeSO_4_ solution (1.52 mg/ml). Furthermore, carbon sources (Table 1, Fig. S1) and antibiotics were added as required (details are provided in each case). For agar plates, 15.0 g/l agar was also added. For construction of fluorescent-labelled strains, we used sterilised LB broth (25.0 g/l Difco LB Broth Miller), YENB broth (7.5 g/l Bacto Yeast Extract and 8.0 g/l Bacto Nutrient Broth), and NA broth (5.0 g/l Bacto Tryptone and 3.0 g/l Bacto Yeast Extract) with antibiotics when needed (details are also provided in each case). Agar media also contained 15.0 g/l agar.

**Table 1:** Carbon sources and their expressions.

**Reagents**	**Expressions**
D-Glucose	Glucose
D-Ribose	Ribose
D-Cellobiose	Cellobiose
D-Raffinose	Raffinose
Glycerol	Glycerol
D-Mannitol	Mannitol
D-Sorbitol	Sorbitol
Sodium Acetate	Acetate
Trisodium Citrate Dihydrate	Citrate
Disodium Succinate Hexahydrate	Succinate
L-Alanine	Alanine
L-Glutamine	Glutamine
L-Isoleucine	Isoleucine
L-Proline	Proline
L-Serine	Serine
Uridine	Uridine

### Bacterial strains

Eight *Pseudomonadota* species were used in this study (Table S2). These species were selected based on their ability to grow in M9-based liquid medium supplemented with either glucose, glycerol, succinate, or proline as a single carbon source at a final concentration of 1.0 mg/ml. Additionally, in order to measure cell density accurately by flow cytometry, we need them to avoid forming aggregates or biofilms in the same medium. Furthermore, to distinguish between the two-species in co-culture, it is necessary to be able to construct strains that express fluorescent protein. We constructed 14 fluorescent-labelled strains by introducing plasmids (pMRE132, pMRE-Tn5-132, pMRE135, and pMRE-Tn5-135) expressing green fluorescent protein (SGFP2) or red fluorescent protein (mScarlet-I) for each species (Supplementary methods S2, Table S3) [39]. To select these transformants, chloramphenicol was used in our experiments. These labelled strains grew distinctly in M9-based liquid medium, which contained 0.5 mg/ml glucose, 0.5 mg/ml succinic acid, and 20 μg/ml chloramphenicol. For abbreviations of bacterial species names, see Fig. 1A.

### Culturing assay

This step was performed using an automated culture system (Biomek NX span-8, Beckman Coulter, California, USA) in a clean booth, which was equipped with a plate reader (FilterMax F5, Molecular Devices, California, USA), plate shaker (StoreX STX44, LiCONiC, Mauren, Liechtenstein), and plate hotel (LiCotel LPX220, LiCONiC) [40]. The incubator was set to 33°C to ensure reliable operation and minimise medium evaporation. First, 14 fluorescent-labelled strains were pre-precultured by inoculating frozen stocks into M9-based liquid medium adjusted to contain 0.5 mg/ml glucose, 0.5 mg/ml succinate, and 20 μg/ml chloramphenicol and incubating for 48 h (200 μl, 96-well plate, 33°C, 500 rpm). Next, we diluted the pre-preculture medium for the next step, pre-culture. This dilution was performed by taking into account the optical density (OD) at 595 nm (OD_595_) and the previously determined growth rates of each strain, in order to ensure that all strains would reach the stationary phase at approximately the same time. The diluted cultures were incubated again for an additional 18 h under the same conditions. After the preculture, the fluorescent cell density was measured by flow cytometry (see Cell density measurement), and then diluted to 2000 cells/μl. For inoculation, 10 μl of a diluted pre-culture was added to 190 μl of medium for each mono-culture, and 5 μl each of two different pre-cultures was added to 190 μl of medium for each co-culture. We assumed that carbon sources in the preculture medium had negligible effects on interspecies interactions due to over 1000-fold dilution. For observing interactions, we cultured each of 14 bacterial strains individually (mono-cultures) and 28 pairs of two species (co-cultures). These experiments were performed across 32 different carbon source environments (Figs 1B and S2). These media included 16 single carbon sources, mixtures of 2, 4, 8, and 16 of carbon sources, and no carbon sources, with total carbon sources concentration adjusted to 1.0 mg/ml and equal contributions when mixed. Here, 20 μg/ml chloramphenicol was not assumed as the carbon sources for growth because it was sufficiently low compared to the other organic compounds. To assess the growth limitations, we performed 14 strains mono-cultures in 16 distinct single-carbon-source environments (Table 1) at 0.5, 1.0, and 1.5 mg/ml. The results showed that for many species and carbon source combinations, the 1.0 mg/ml carbon sources were the limiting factors for growth (Fig. S3). Competitive interactions may be more frequently observed under carbon-limited conditions than in carbon-rich environments, because the depletion of available resources is expected under carbon limitation. All cultures were shaken for 72 h in which many combinations were saturated, with measuring OD every 3 h (200 μl, 96-well plate, 33°C, 500 rpm). After 72 h, cultures were stored at −80°C for fluorescent cell density measurement.

To investigate the effect of plasmid-based labelling on bacterial growth, we examined the relationship between fluorescence intensity and the number of carbon sources to assess the environmental dependence of plasmid copy number (Supplementary methods S3). As a result, no significant correlation was observed between the average fluorescence intensity and carbon source diversity for either the SGFP2 fluorescent-labelled strain or the mScarlet-I fluorescent-labelled strain in single cultures (Spearman’s rank correlation: $\rho =0.02,P=.66$ for SGFP2 fluorescent cell and Spearman’s rank correlation: $\rho =0.01,P=.77$ for mScarlet-I fluorescent cell). Furthermore, using data from previous studies employing the same plasmid [32], we analysed co-culture results between fluorescent-labelled and unlabelled strains of the same species to assess the cost of plasmid maintenance (Supplementary methods S3). Because the unidirectional effect (${E}_m^{i,i}$) of unlabelled strains on fluorescent-labelled strains can reflect the potential cost associated with plasmid maintenance, we examined whether this effect correlated with the average interaction types (${\overline{\theta}}_m$), which were obtained by averaging interaction types across the 16 single-carbon-source environments used in this study. Here, ${E}_m^{i,i}$ and ${\overline{\theta}}_m$ are defined in Quantification of the interaction types, and ${E}_m^{i,i}$ is expected to become smaller when the cost is higher. The analysis, however, revealed no significant correlation (Spearman’s rank correlation: $\rho =0.07,P=.26$). Based on these analyses, we assumed that plasmid-based labelling does not have a substantial impact on the assessment of interspecies interactions.

### Cell density measurement

To measure fluorescent cell density, a portion of culture medium was mixed with an appropriate amount of standard fluorescent particle solution (Fluoresbrite Polychromatic Red Microspheres 1.0 μm, Polysciences, Pennsylvania, USA) and PBS (KH_2_PO_4_ 144 μg/ml, NaCl 9000 μg/ml, Na_2_HPO_4_ 421 μg/ml, pH 7.4), with the concentration of fluorescent particles known. Information on each particle in each diluted sample was collected by flow cytometry (FACS Aria III Cell Sorter, BD, New Jersey, USA; flow rate: 1.0, measurement time: 1 min). Fluorescence information at 530 ± 15 nm wavelength emitted by the excitation light at 488 nm (green fluorescence in Fig. S4) and at 582 ± 7.5 nm wavelength emitted by the excitation light at 561 nm (red fluorescence in Fig. S4) were used for strains identification. The fluorescence information at 670 ± 7 nm wavelength emitted by the excitation light at 561 nm wavelength and the side scatter information were also used to identify the standard fluorescent particles. The cell density for each of the two fluorescent-labelled cell types was obtained by calculating the relative concentration to the standard fluorescent particles. The cell density of green or red fluorescent cells was set to 4.57$\times$10^5^ cells/ml if it was lower than this threshold. This threshold was determined based on the 90th percentile of the total green and red fluorescent cell density observed in co-culture and mono-culture without carbon sources.

### Identification of the interaction classes

The interspecies interactions were classified for each combination of growth environment and bacterial pair. Following the method used in a previous study [30], we identified two unidirectional effects that occurred between the two species in the co-culture and classified the bidirectional interaction classes based on the combination of the two unidirectional effects. Specifically, we calculated the ratio of the fluorescent cell density of focal bacterial species in the co-culture at 72 h to the average fluorescent cell density of the same species in mono-culture with the same carbon sources. This ratio is expressed as $\frac{Y_m^{i,j}(r)}{{\overline{Y}}_m^i}$, where ${Y}_m^{i,j}(r)$ represents the fluorescent cell density for bacterial species $i$ at 72 h when the $r$-th replicate was co-cultured with bacterial species $j$ in growth environment $m$ ($r\in \left\{1,2,3\right\}$), and ${\overline{Y}}_m^i$ indicates the average cell density when focal species $i$ was mono-cultured in the same environment $m$. Next, two-tailed t-tests were performed on the null hypothesis that the average of these values is equal to 1. Then, a multiple comparison using the Benjamini–Hochberg method [41] was conducted (false discovery rate [FDR]: 0.05). When the average of these ratios was significantly greater than 1, the unidirectional effect was considered positive (facilitated, +); less than 1, negative (inhibited, −); and when the null hypothesis was not rejected, it was considered to have no effects (unaffected, 0). Finally, the bidirectional interaction classes were categorised according to a widely recognised classification [5–7] based on the unidirectional effects (Fig. 1C). According to this classification, a high cell density in co-culture does not imply facilitation if the mono-culture density is similarly high, nor does a low co-culture density necessarily indicate inhibition if the mono-culture density is also low.

### Quantification of the interaction types

Synergism degree of interspecies interactions was quantitatively assessed as “interaction types”, based on the method used by previous study [32]. Interactions between bacterial species $i$ and $j$ in growth environment $m$ were evaluated using equations (1) and (2):


(1) \begin{equation*} {E}_m^{i,j}={\log}_2\frac{{\overline{Y}}_m^{i,j}}{{\overline{Y}}_m^i} \end{equation*}

(2) \begin{equation*} {\theta}_m^{i,j}=\left\{\begin{array}{@{}l}\arctan \left(\frac{E_m^{i,j}+{E}_m^{j,i}}{\ \left|{E}_m^{i,j}-{E}_m^{j,i}\right|\ }\right)\frac{180}{\mathrm{\pi}}\ \left[{}^{{}^{\circ}}\right],\mathrm{if}\ {E}_m^{i,j}\ne{E}_m^{j,i}\\{}0\kern9.5em \left[{}^{{}^{\circ}}\right],\mathrm{if}\ {E}_m^{i,j}={E}_m^{j,i}=0\end{array}\right. \end{equation*}

where ${\overline{Y}}_m^{i,j}$ represents the average fluorescent cell density of species $i$ at 72 h of triplicate co-cultures with bacterial species $j$ in growth environments $m$ and ${\overline{Y}}_m^i$ represents the average fluorescent cell density of species $i$ at 72 h of triplicate mono-cultures in growth environment $m$. ${E}_m^{i,j}$ quantifies the unidirectional effects on species $i$ when co-cultured with species $j$ in the growth environment $m$. Similarly, ${E}_m^{j,i}$ quantifies the unidirectional effects on species $j$. Then, we plot the smaller of these two values, $\min ({E}_m^{i,j},{E}_m^{j,i})$, on the horizontal axis and the larger of these two values, $\max ({E}_m^{i,j},{E}_m^{j,i})$, on the vertical axis in the Cartesian plane. In equation (2), ${\theta}_m^{i,j}$ represents the angle $(-{90}^{{}^{\circ}}\le{\theta}_m^{i,j}\le{90}^{{}^{\circ}})$ that the line segment connecting this plotted point and the origin makes with the line where $\max ({E}_m^{i,j},{E}_m^{j,i})=-\min ({E}_m^{i,j},{E}_m^{j,i})$ (dotted line in Fig. 1D), and was taken as a quantitative measure of the interaction types. A higher ${\theta}_m^{i,j}$ indicates a more synergistic interaction, whereas lower ${\theta}_m^{i,j}$ reflect more competitive interactions. When all values of ${\overline{Y}}_m^i$, ${\overline{Y}}_m^j$, ${\overline{Y}}_m^{i,j}$ and ${\overline{Y}}_m^{j,i}$ were at the lower limit of 4.57$\times$10^5^ cells/ml, ${\theta}_m^{i,j}$ was set to ${0}^{{}^{\circ}}$. Furthermore, we calculated two forms of the average interaction types ${\overline{\theta}}_m$ and ${\overline{\theta}}^{i,j}$. Here, ${\overline{\theta}}_m$ is defined as the mean of ${\theta}_m^{i,j}$ observed in the same environment (i.e., ${\overline{\theta}}_m=\frac{1}{{}_8{}{\mathrm{C}}_2}\sum_{i,j}{\theta}_m^{i,j}$; the mean was calculated across all pairwise combinations of the eight microbial species), and ${\overline{\theta}}^{i,j}$ is calculated as the mean of ${\theta}_m^{i,j}$ observed in the same bacterial pair (i.e., ${\overline{\theta}}^{i,j}=\frac{1}{16}\sum_m{\theta}_m^{i,j}$; the mean was calculated across all 16 single-carbon-source environments).

### Analysis of mixing carbon sources impact

The effect of mixing carbon sources on bacterial growth was investigated using yield indicators, cell density or OD. This effect was examined by comparing Group 1, which represents growth in multi-carbon-source environments, with Group 2, which represents the average of growth when each carbon source is provided individually, as follows:


\begin{align*} \left\{{\log}_{10}{Y}_{1,2,\dots, n}^i(r)\ \big|r=1,2,3\right\} \end{align*}

\begin{align*} &\left\{\frac{\sum_{k=1}^n{\overline{\log_{10}Y}}_k^i}{n}+\sqrt{\frac{\sum_{k=1}^n{\left({u}_k^i\right)}^2}{n}},\frac{\sum_{k=1}^n{\overline{\log_{10}Y}}_k^i}{n},\right. \\ &\quad\left. \frac{\sum_{k=1}^n{\overline{\log_{10}Y}}_k^i}{n}-\sqrt{\frac{\sum_{k=1}^n{\left({u}_k^i\right)}^2}{n}}\right\} \end{align*}

where ${Y}_{1,2,\dots, n}^i(r)$ represents the yield in the $r$-th mono-culture of species $i$ with a mixture of carbon sources 1, 2, ..., $n$. Each ${\overline{\log_{10}Y}}_k^i$ represents the average log_10_ yield in the triplicate mono-cultures of species $i$ with carbon source $k$, and ${\left({u}_k^i\right)}^2$ represents the unbiassed variance of the log_10_ yield, reflecting the variability in the mono-culture experiments. Thus, Group 1 represents an actual population of log_10_-transformed values of measured cell density in multiple carbon sources, whereas Group 2 refers to a theoretical population of log_10_-transformed values whose mean corresponds to the average cell density obtained when each carbon source is supplied individually, with an appropriate variance. To check for significant changes in the means for these two groups, two-tailed t-tests were performed and evaluated (FDR: 0.05).

### Statistical analyses of interspecies interactions

To explore the determinants of the interspecies interactions, several statistical analyses were performed. The raw data and analysis codes for R (version: 4.2.2) [42] are available on Zenodo [43] and GitHub (https://github.com/HirokiOnoGit/Carbon-sources-shape-bacterial-relations). To find out whether the interaction classes varied significantly by single carbon source or bacterial pair, Chi-square tests [44] were performed. These tests were performed by grouping the interaction classes by each carbon source or bacterial pair and examining whether the differences were by chance or not in all pairwise cases. Pairwise permutational multivariate analysis of variance (PERMANOVA) [45] was performed using vegan package [46] to examine the relationship between the observed interactions and the biochemical categories of carbon sources. This analysis was carried out by dividing the carbon sources into sugars (monosaccharides, disaccharides, trisaccharides), sugar alcohols, carboxylate ions, amino acids, and nucleic acids to see if the interaction classes observed in each were significantly biassed compared to the full data. We also performed applied gene set enrichment analysis (GSEA) [47] using clusterProfiler package [48], which is widely used for gene expression analysis, to identify biases in the interaction classes likely to form in each bacterial species. This analysis was based on examining the bias in the average rank of the interaction type for each bacterial pair. Furthermore, to investigate the relationship between the bacterial growth potential and interspecies interactions, we performed clustering analysis for each bacterial species using Ward’s method [49]. This analysis utilised average cell densities obtained from mono-culture experiments across 32 environmental conditions to classify bacterial species–environment combinations as “growers” or “non-growers”. Based on this classification, we performed an enrichment analysis to assess whether each interaction class was significantly overrepresented in each case. This analysis focused on environments containing one, two, or four carbon sources, identifying cases in which both species were classified as growers, or where at least one was a non-grower. The clusterProfiler package [48] was used for this analysis. Further details are described in the supplementary information (Supplementary methods S3–S7).

## Results

### Various interaction classes were observed in single-carbon-source environments

In this study, we analysed bacterial co-cultures in laboratory environments to explore the relationship between carbon source diversity and interspecies interactions. We used eight bacterial species isolated from various environments (Table S2), each labelled with either green (SGFP2) or red (mScarlet-I) fluorescent proteins [39], and paired them in combinations (Figs 1A, B, and S2A). Cultures were grown in M9-based liquid media with 31 distinct carbon source compositions derived from 16 organic compounds, including sugars, alcohols, carboxylates, amino acids, and nucleic acids. These carbon sources were selected based on previous studies [32] and, due to operational constraints, were randomly combined, with up to 16 carbon sources per mixture (Fig. S2B). The total concentration of carbon sources was set at 1.0 mg/ml, which was found to be a level that restricted growth in many combinations of strains and single carbon sources (Fig. S3). When multiple carbon sources were included, they were mixed in equal amounts by mass. Additionally, cultures without any carbon source were included as controls. We conducted both mono-cultures and two-species co-cultures, measuring OD_595_ every 3 h with a plate reader. After 72 h, a timepoint sufficient for growth saturation in most combinations, fluorescent cell density was measured using flow cytometry. This allowed us to classify and quantify interspecies interactions statistically by comparing the growth of mono- and co-cultures under the same environmental conditions, as detailed in Materials and methods (Fig. 1C and D).

**Figure 2:**
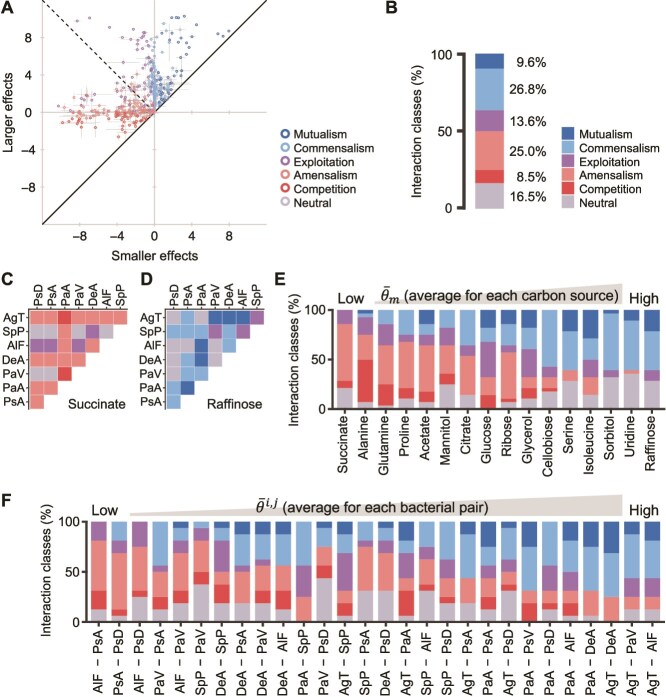
Diverse interspecies interactions were observed in single-carbon-source environments. (A) Interspecies interactions for 28 bacterial pairs across 16 single-carbon-source environments. The smaller effects ${\min}\left({{E}}_{{m}}^{{i},{j}},{{E}}_{{m}}^{{j},{i}}\right)$ are plotted on the horizontal axis, whereas the larger effects ${\max}\left({{E}}_{{m}}^{{i},{j}},{{E}}_{{m}}^{{j},{i}}\right)$ are plotted on the vertical axis. Colours represent different interaction classes. Error bars represent the standard deviation across biological triplicates. (B) Percentage bar chart of interaction classes observed in single-carbon-source environments, corresponding to data in panel A. (C, D) Classification of interspecies interactions when succinate (C) or raffinose (D) was provided as the single carbon source. Colours at the intersections of the vertical (species ${i}$) and horizontal (species ${j}$) axes represent the interaction classes between species ${i}$ and ${j}$. (E, F) Interaction classes separated by carbon sources (E) or bacterial pairs (F). Bars in panel E are ordered by average interaction types ${\overline{{\theta}}}_{{m}}$ for each carbon source, whereas bars in panel F are ordered by average interaction types ${\overline{{\theta}}}^{{i},{j}}$ for each bacterial pair. See Fig. 1A for abbreviations of bacterial species names.

In single-carbon-source environments, various interaction classes were observed (Fig. 2A and B). The observed interaction classes were categorised as follows: synergistic interactions (mutualism and commensalism) accounted for 36.4%, exploitative interactions (exploitation) for 13.6%, competitive interactions (competition and amensalism) for 33.5%, and no interactions (neutral) for 16.5%. To reveal the factors that contributed to this wide variety of interspecies interactions, we then recapitulated observed interactions for each growth environment and bacterial pair as described below.

The interspecies interactions were significantly influenced by the provided carbon sources (Chi-square tests [44], $P=1\times{10}^{-5}$ [Monte Carlo estimated, ${10}^5$ iterations]; Table S4). To quantify interactions, we used the interaction types ${\theta}_m^{i,j}$ [32], representing the degree of synergism for each bacterial pair comprising species $i$ and $j$ in the growth environment $m$ (Fig. 1D, Material and methods). In brief, interaction types ${\theta}_m^{i,j}$ range from $-{90}^{{}^{\circ}}$ to ${90}^{{}^{\circ}}$, with higher values indicating more synergistic interactions. When ${\theta}_m^{i,j}$ is $-{90}^{{}^{\circ}}$, both strains inhibit the growth of each other equally, whereas when ${\theta}_m^{i,j}$ is ${90}^{{}^{\circ}}$, both strains facilitate each other equally. Sorting the carbon sources by the average interaction types ${\overline{\theta}}_m$, defined as the mean of ${\theta}_m^{i,j}$ observed in the same environment, revealed that succinate promoted the most competitive interactions (Fig. 2C, leftmost column in Fig. 2E, ${\overline{\theta}}_m=-{30.3}^{{}^{\circ}}$), whereas raffinose led to the most synergistic interactions (Fig. 2D, rightmost column in Fig. 2E, ${\overline{\theta}}_m={48.7}^{{}^{\circ}}$). Previous research on genome-wide metabolic modelling has suggested that the biochemical category of carbon sources is one of the crucial factors explaining differences in bacterial interaction classes [22]. By contrast, the previous study [32] pointed out that the biochemical category may not always explain interactions as well as previously expected. Given this, we performed a PERMANOVA on our data, following the previous study and found no significant differences in interaction patterns within or between biochemical categories (FDR: 0.05; Table S5, Fig. S5). Additionally, hierarchical clustering of all interactions across bacterial pairs and carbon sources showed no clear grouping based on biochemical categories (Fig. S6). These findings suggest that the observed differences in interspecies interactions across different carbon sources are not driven by the biochemical category of carbon sources alone.

Interspecies interactions varied depending on bacterial pairs. When comparing the average interaction types ${\overline{\theta}}^{i,j}$, calculated as the mean of ${\theta}_m^{i,j}$ observed in the same bacterial pair, *Alcaligenes faecalis* and *Pseudomonas asiatica* (AlF-PsA) formed the most competitive interactions (${\overline{\theta}}^{i,j}=-{22.7}^{{}^{\circ}}$), whereas *Agrobacterium tumefaciens* and *A. faecalis* (AgT-AlF) formed the most synergistic interactions (${\overline{\theta}}^{i,j}={36.2}^{{}^{\circ}}$) (Fig. 2F). Chi-square tests confirmed that bacterial pairs significantly influenced interaction classes (Chi-square tests [44], $P=1.1\times{10}^{-4}$ [Monte Carlo estimated, ${10}^5$ iterations]; Table S6). Nevertheless, many bacterial pairs displayed various interactions, ranging from competitive to synergistic, depending on the provided carbon sources (Fig. 2F). To determine whether certain species were predisposed to form synergistic or competitive interactions, we adapted the framework of GSEA [46]—originally developed for gene expression data—to our bacterial pair-interaction dataset (FDR: 0.05; Table S7, Fig. S7). No specific species were found to exhibit a significant bias toward either synergistic or competitive interactions, suggesting that interaction outcomes are affected by complex factors beyond species identity alone. Taken together, our findings indicate that both carbon sources and bacterial pairs influence interspecies interactions.

### Increasing the number of carbon sources led to more competitive interactions

**Figure 3:**
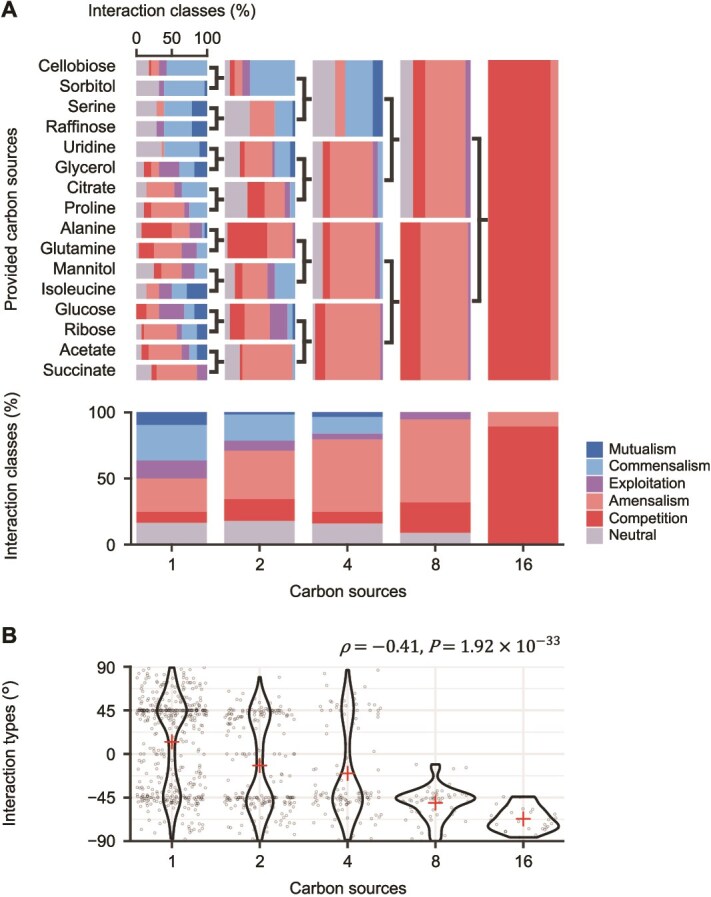
Relationship between carbon source diversity and bacterial interspecies interactions. (A) Percentage bar charts showing interaction classes in each environment. The upper panel displays horizontal bar charts categorised by provided carbon sources (rows) and the number of carbon sources (columns). Two-way symbols indicate that the carbon sources on the left were mixed and provided as a combination to the right. The lower panel presents vertical bar charts summarising interaction classes based on the number of carbon sources. The leftmost 16 horizontal bars are replots of Fig. 2E, whereas the leftmost vertical bar aligns with Fig. 2B. The datasets used for the rightmost horizontal and vertical bars are identical. (B) Violin plots with jittered points showing a negative correlation between the number of carbon sources and the interaction types ${{\theta}}_{{m}}^{{i},{j}}$. Cross-shaped points represent the average interaction types for each number of carbon sources. Spearman’s rank correlation (*ρ*) and its associated p-value (*P*) are shown in the top right corner.

To clarify how carbon source diversity affects bacterial interactions, we next examined the influence of increasing the number of carbon sources per medium. Carbon sources were mixed in equal mass amounts, set to a total concentration of 1.0 mg/ml, consistent with single-carbon-source environments. The supplementary data (Table S8, Fig. S8) present all interspecies interactions across different carbon source and bacterial pair combinations. When categorising these results by number of carbon sources, we found that 33.5% of interactions were competitive in single-carbon-source environments (bottom row and leftmost column in Fig. 3A), whereas all interactions were competitive when the number of carbon sources reached 16 (bottom row and rightmost column in Fig. 3A). Furthermore, we confirmed that correlation between the number of carbon sources and the interaction types ${\theta}_m^{i,j}$ was negative (Spearman’s rank correlation: $\rho =-0.41,P=1.92\times{10}^{-33}$; Fig. 3B). Based on these observations, we concluded that bacterial interactions tended to be more competitive as carbon source diversity increased.

### Competition is more frequent in environments where both species can grow

To understand how an increased carbon source diversity led to more competitive interactions, we analysed the observations in detail. Potential mechanisms of competitive interactions include, e.g. resource competition [33–36] and the production of growth inhibitors [50, 51]. In particular, in our set-up, the amount of provided carbon sources was found to be a growth constraint for most bacteria and carbon source combinations (Fig. S3). This leads us to speculate that the contest for carbon sources contributes to a major mechanism of competitive interactions.

**Figure 4:**
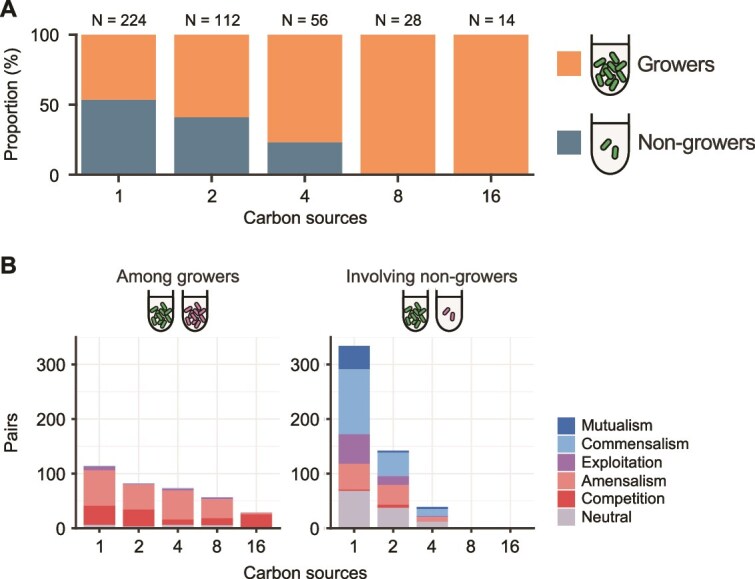
Competition is more frequent in environments where both species are growers. (A) Relationship between the number of carbon sources and growth potential in mono-cultures. Mono-cultures were categorised into “growers” or “non-growers” for each species based on growth yields, as detailed in the main text. Bar charts show the proportion of “growers” and “non-growers” for each number of carbon sources. (B) Influence of the growth potential on interspecies interactions. Bar charts show the distribution of interaction classes in environments where both species were “growers” (left) and where at least one species was a “non-grower” (right). Colours represent different interaction classes.

We hypothesised that increasing the number of carbon sources results in greater availability of carbon sources, which in turn leads to more frequent competitive interactions due to the intensified contest for available resources. We tested this hypothesis by evaluating two related sub-hypotheses: (i) increasing the number of carbon sources improves resource utilisation and allows more bacterial species to grow, and (ii) competitive interactions occur more frequently between species that are both able to grow. To test the sub-hypothesis (i), we first calculated ${\overline{Y}}_m^i$, the average cell density of bacterial species $i$ cultured in triplicate under growth environment $m$. The distribution of ${\overline{Y}}_m^i$ for each carbon source naturally formed two distinct clusters—one corresponding to species that showed substantial growth (“growers”) and the other to species with little to no growth (“non-growers”). Given these data characteristics, we applied Ward’s clustering method [49] to distinguish between growers and non-growers (Fig. S9). Finally, we examined how the proportion of growers varied with the number of carbon sources, and found that environments with more carbon sources tended to support a higher number of growers (Figs 4A and S10). Next, to evaluate the sub-hypothesis (ii), we examined the interspecies interactions between species that are both growers. We divided the bacterial interactions into two groups: those that occurred amongst growers, and those that occurred when at least one species was a non-grower. The results showed that competitive interactions accounted for more than 82% of the interspecies interactions when both species were growers (Fig. 4B left), regardless of the number of carbon sources (83.7%, 88.3%, 82.9%, 85.7%, and 100.0% when the carbon sources were 1, 2, 4, 8, and 16 species, respectively). By contrast, in interactions involving at least one non-grower species, a variety of interspecies interactions were formed, ranging from synergistic to competitive (Fig. 4B right). In order to statistically investigate whether specific interaction classes significantly accumulated in these two groups, we performed an enrichment analysis. We analysed the data corresponding to environments with one, two, and four carbon sources, where both growers and non-growers were identified, and found that competitive interactions were significantly enriched in interactions between growers (FDR: 0.05; Table S9, S10). Our analysis method did not detect competitive interactions when the average cell density in mono-culture is below 4.57$\times$10^5^ cells/ml, which is the lower limit in our quantification. To rule out this methodological limitation, we performed the same analysis, excluding combinations where the average cell density in mono-culture is lower than this limit. The results confirmed that even after excluding these combinations, competition was still frequent between growers (Fig. S11).

### Growth in multi-carbon-source environments generally matches or exceeds the average growth when each carbon source is provided individually

Bacterial growth can be facilitated by different scenarios as carbon source diversity increases. In this study, we varied the number of carbon sources while keeping the total amount constant at concentrations expected to be fully consumed in most combinations. Under this condition, a straightforward expectation is that the growth yield of multi-carbon-source environments would be consistent with the average yield before mixing the corresponding single-carbon-source environments. Alternatively, some carbon source combinations may enhance growth by fulfilling nutrient requirements [52, 53], whereas others could suppress it due to carbon source toxicity [50, 51].

**Figure 5:**
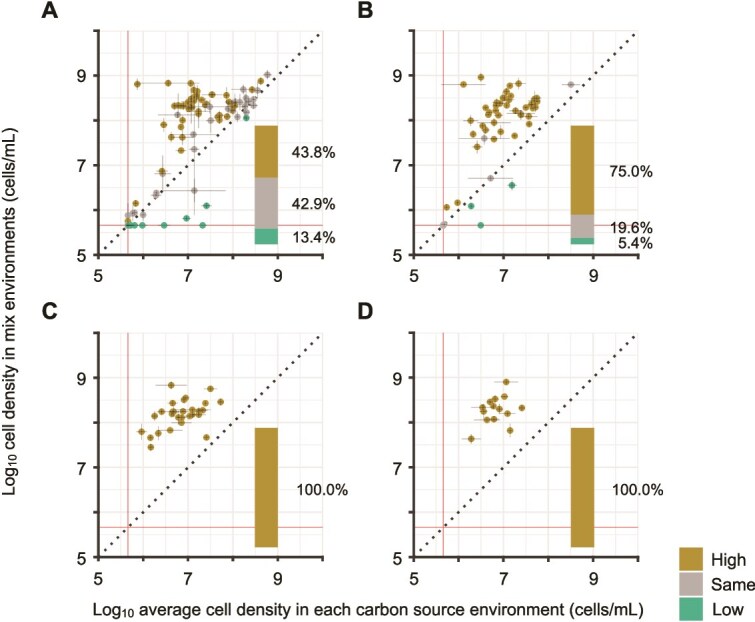
Relationship between growth yields in multi-carbon-source environments and the average yields in the corresponding single-carbon-source environments. (A–D) The vertical axis represents the log_10_ fluorescent cell densities of species ${i}$ in mono-cultures under multi-carbon-source environments, whereas the horizontal axis represents the average log_10_ fluorescent cell density of the same species in mono-cultures when provided with each carbon source individually. The dashed lines represent the points where the values on the vertical and horizontal axes are equal. The coloured horizontal and vertical lines represent the lower limit of cell density. Colour of each point denotes three scenarios based on whether the vertical axis values are higher than, not significantly different from, or lower than the horizontal axis values. The colours of each point were determined by statistical comparisons with multiple testing correction (see Materials and methods). Insets show the distribution of these three scenarios. Panels A–D correspond to environments containing 2, 4, 8, and 16 carbon sources, respectively.

To reveal how increasing carbon source diversity influences bacterial growth, we compared growth yields in multi-carbon-source environments to those in the corresponding single-carbon-source environments. In two-carbon-source environments, we first calculated the average yield of bacterial species $i$ cultured in a mixed environment with two carbon sources $m$ and $n$ across biological triplicates, denoted as ${\overline{Y}}_{m,n}^i$. We then compared ${\overline{Y}}_{m,n}^i$ with the average yields of the same bacterial species in the corresponding single-carbon-source environments, ${\overline{Y}}_m^i$ and ${\overline{Y}}_n^i$. In 86.7% of cases, ${\overline{Y}}_{m,n}^i$ matched or exceeded the mean of ${\overline{Y}}_m^i$ and ${\overline{Y}}_n^i$ (two-tailed t-test, FDR: 0.05; Figs 5A and S12A). Similarly, when 4, 8, and 16 carbon sources were provided, growth yields in multi-carbon-source environments generally matched or exceeded the average of the yields in the corresponding single-carbon-source environments (Figs 5B–D and S12B–D).

To confirm that our results were not biassed by environments in which carbon sources were not fully consumed, we repeated the analysis after excluding such cases. First, we identified these environments by comparing growth yields across different carbon source concentrations in single-carbon-source conditions (Fig. S3). Then, we excluded data from combinations where 1.0 mg/ml carbon sources were likely not fully consumed and repeated the analysis. The results derived from the refined dataset remained consistent with the previous results, indicating that incomplete carbon source consumption did not significantly influence the outcomes (Fig. S13). Overall, we conclude that bacterial growth in multi-carbon-source environments generally matches or exceeds the average growth in corresponding single-carbon-source environments. Thus, both simple additive effects and synergistic growth enhancement contribute to the facilitation of bacterial growth as carbon source diversity increases, likely leading to the processes occurring in environments that support bacterial growth, such as resource competition [33–36] or quorum sensing [54, 55].

### Similarity of metabolic networks and numbers of possible carbon-containing metabolic by-products partially explain interaction types

The analyses comparing bacterial growth in multi-carbon-source environments with that in the corresponding single-carbon-source environments suggest that growth potential has a strong influence on interspecies interactions. Previous studies have also indicated that the similarity of metabolic networks [32] and the number of possible carbon-containing metabolic by-products [56] are major factors affecting interspecies interactions. Therefore, we returned to the analysis of interspecies interactions observed in single-carbon-source environments to examine these influences. In the previous study [32], “metabolic distance” was defined based on bacterial growth data from 33 distinct carbon sources and found a positive correlation with average interaction types (Spearman’s rank correlation: $\rho =0.75,P<.01$). Following their approach, we calculated metabolic and phylogenetic distances (Fig. S14) and assessed their relationships with interaction types (Supplementary methods S4 and S5). Our results revealed that correlations between metabolic (or phylogenetic) distance and interaction types were weaker compared to previous studies (Figs S15 and S16). For instance, the correlation between metabolic distance and interaction types was weaker (Spearman’s rank correlation: $\rho =0.07,P=7.13\times{10}^{-3}$), and, following the approach of previous study [32], no significant correlation was found when averaging the interaction types across bacterial pairs (Spearman’s rank correlation: $\rho =0.16,P=.41$).

To gain further insight into factors determining interaction types, we examined the influence of the number of possible carbon-containing metabolic by-products. For this purpose, we analysed constraint-based metabolic models constructed with CarveMe [57], a genome-scale metabolic model reconstruction tool. For constructing these models, we used publicly available genome information (Table S11) and growth information in single-carbon-source environments (Fig. S1) for the eight bacterial species. We performed flux balance analysis (FBA) to simulate metabolic flux distributions optimised for biomass production under each single-carbon-source environment [56, 58]. From these distributions, we identified metabolites containing carbon atoms and associated with reactions carrying nonzero flux, and we defined these as possible carbon-containing metabolic by-products (Supplementary methods S6). Because an FBA solution represents just one of multiple possible flux distributions, we restricted our analysis to by-products associated with reactions essential for optimal growth—specifically, reactions whose removal decreased biomass production by more than 1%. This stringent criterion enabled us to estimate the minimal number of by-products required under optimal conditions. Additionally, we confined the analysis to cases where substantial population growth (4.57 $\times$ 10^5^ cells/ml or higher) was experimentally verified. As a result, we obtained the relationship between the number of possible metabolic by-products and carbon sources for each species (Fig. S17). Isoleucine and uridine produced relatively large metabolic by-product pools in most bacterial species capable of utilising these carbon sources. Raffinose, utilised by only *Sphingomonas paucimobilis* (SpP), yielded the largest predicted by-product pool amongst all carbon sources tested for this species. These carbon sources are metabolised through multiple steps before entering the central metabolic pathways and give rise to both core metabolic intermediates and additional compounds that are not typically generated by gluconeogenic substrates (e.g. tricarboxylic acid cycle or lower glycolysis intermediates). This results in a broader range of metabolic by-products with the potential for cross-feeding, consistent with the previous observation [56]. Despite these examples, correlation analysis between the rank of ${\overline{\theta}}_m$ and the predicted number of by-products revealed that significant positive correlations were rare across species. This finding aligns with the fact that ${\overline{\theta}}_m$ reflects not only synergistic interactions, but also competitive ones, which may obscure the specific contribution of cross-feeding. Taken together, these analyses support the notion that the interspecies interactions observed in our study may, at least partially, reflect both resource competition [33–36] and cross-feeding of carbon-containing metabolic by-products [56, 59].

## Discussion

In this study, we explored bacterial interactions under different environmental conditions and identified that competitive interactions are more frequent in environments with higher carbon source diversity (Fig. 3). To understand how an increase in carbon source diversity was associated with more frequent competitive interactions, we tested two sub-hypotheses (i) increasing the number of carbon sources improves resource utilisation and allows more bacterial species to grow, and (ii) competitive interactions occur more frequently between species that are both able to grow. Our experimental data aligned with these two sub-hypotheses, supporting the previous assertion [32] that competitive interactions are more likely in environments conducive to bacterial growth (Fig. 4). Along with these scenarios, we also found that combining multiple carbon sources enhanced bacterial growth in mono-cultures, either matching or exceeding the levels observed with individual carbon sources, indicating that greater carbon source diversity promotes bacterial growth (Fig. 5) [60, 61]. This emergent growth improvement might lead to more frequent interspecies competition in co-cultures under higher carbon source diversity. In conclusion, whereas previous studies have focused on the impact of carbon source diversity on the species composition of bacterial communities [56], our findings highlight its impact on the types of bacterial interspecies interactions and offer insights into understanding bacterial communities under complex environments containing diverse carbon sources.

Bacterial interspecies interactions are influenced by both environmental conditions and bacterial pairs. Predominance in competitive interspecies interactions is widely observed in binary bacterial communities under complex environmental conditions containing diverse carbon sources [26–30]. In contrast, our approach, which examined interactions across environments with varying numbers of carbon sources, indicates that synergistic interactions can also frequently occur depending on the environment. Although these findings align with previous studies using binary strain communities, we also noticed some quantitative differences. For example, the previous study [32] reported that synergistic interactions accounted for 17% in single-carbon-source environments, whereas we found a significantly higher proportion of 36.4% (Fig. 2B). This discrepancy may be attributed to differences in the taxonomic diversity of bacterial species. We used a broader range of species spanning three classes, whereas the previous study focused on species within a single class (Fig. S14) [32]. Because closely related species with similar metabolic networks are expected to engage in competitive interactions [33–36], the inclusion of distantly related species in our study likely resulted in fewer competitive interactions and a relative increase in synergistic interactions. These differences highlight the potential influence of taxonomic diversity on observed interaction types. In particular, the relatively high taxonomic diversity in this study also provides new insights into how bacterial combinations influence interspecies interactions. The previous study reported a positive correlation between “metabolic distance” and average interaction types [32]. However, in our dataset including more cross-class pairs, this correlation was not observed (Figs S15 and S16). These results suggest that whereas metabolic or phylogenetic distances can explain interactions within a class, where resource competition dominates, they might be insufficient for predicting interactions across classes, where synergism occurs more frequently [62].

Although our study provides crucial insights into the determinants of bacterial interactions, several limitations should be acknowledged. First, our experiments do not reveal the molecular mechanisms of the bacterial interactions. However, some previous studies are helpful in estimating them; e.g. bacterial growth facilitation is known to be triggered by processes such as the exchange of metabolites [22, 63] and secretion of enzymes [64]. Regarding growth inhibition, it could stem from resource competition [33–36] and the secretion of inhibitory substances such as organic acids and antibiotics [50, 51]. Moreover, quorum sensing between closely related species can facilitate or inhibit interspecific growth [54, 55]. We consider that the observed interactions reflect the net result of these processes occurring simultaneously. The relative contribution of these processes will vary according to environmental conditions and bacterial pairs. In order to reveal the details of specific molecular mechanisms, it would be useful to understand these processes in relation to the observed interactions, e.g. by omics analysis of transcripts and metabolites.

A second limitation concerns our interpretation of synergistic effects of growth in multi-carbon-source environments. We observed that increasing the number of carbon sources led to enhanced bacterial growth, exceeding the average growth observed in individual carbon source environments (Figs 5, S12, and S13). These phenomena can be attributed to several mechanisms described below: the fulfilment of nutritional requirements [52, 53], the alleviation of specific metabolic stresses [65], and the optimisation of the metabolic network [66]. Each of these mechanisms may arise from increasing carbon source diversity and, in turn, enhance bacterial growth. Additionally, the effects due to mixing carbon sources have been actively studied as a phenomenon known as the priming effect [60, 61]. Although our study does not precisely identify the mechanisms, these previous studies may help elucidate the processes driving these synergistic effects.

A third limitation of our study is the temporal dynamics of interactions were not fully addressed. Although we assessed interactions at 72 h after the start of cultivation, when most combinations had reached their steady-state yield, the outcomes of interaction assessments may vary depending on the timing of observation [67]. Nevertheless, we consider that our approach provides meaningful insight, as it captures interaction patterns under well-controlled and contrasting environmental conditions, after sufficient growth to reach stationary phase. This snapshot allows for systematic comparisons across a large number of conditions, while minimising confounding factors related to differences in growth stage. High-throughput approaches that track interaction dynamics over time [68] will be important in the future, but our study contributes complementary data by focusing on the outcomes after standardised cultivation. In addition, our method differs from chemostat or serial passaging cultures, in which extinctions are more likely to occur. Our methodology allows for the assessment of species combinations even if they would not coexist under prolonged cultivation conditions, whereas it fundamentally differs from long-term experiments aimed at evaluating species coexistence [56]. Given these methodological differences, it should be mentioned that it is not always appropriate to predict the results of this study by the competitive exclusion principle [69], which posits that only niche-dominant species can coexist. A promising direction for future research would be investigating how the composition of the combinations observed in this study would change if observations were extended under conditions that permit species extinction over time.

A further issue to consider is whether the patterns observed in this and previous studies can be generalised to other environments or phylogenetic groups. In our experiments, we used relatively simple carbon sources, including compounds from the tricarboxylic acid cycle. This restriction may bias results in competition compared to macromolecules, which are expected to lead to cross-feeding [56, 59]. Similarly, we selected the bacteria observed in this study to satisfy the following two requirements: (i) the ability to grow in M9-based liquid medium supplemented with one of the following carbon sources—glucose, glycerol, succinate, or proline without visible aggregation; (ii) the capacity for transformation (Supplementary methods S2). Due to these selection criteria, our experimental data probably do not capture the interspecies interactions that take place in biofilms, which are thought to be frequently synergistic [70]. Our findings suggest that such culturable bacteria, which are expected to have access to a wider range of carbon sources [71–73], are prone to competitive interactions. This indicates that competitive interactions are less common in communities containing unculturable bacteria. Natural environments, such as the gut [74] and soil [75, 76], harbour thousands of bacterial species across multiple phyla with highly diverse carbon sources and are much more ecologically and phylogenetically complex than the in vitro systems used in this study. Our results show that frequent synergistic interactions in single-carbon-source environments, such as those observed by the previous study [32], may differ substantially from interactions in multi-carbon-source environments, suggesting that further investigations are needed to understand the interaction patterns in complex natural environments. In addition, because our study assessed interactions using a standardised inoculum and carbon source concentration, our understanding is also limited with regard to interactions under other conditions. For example, if unequal amounts of bacteria are inoculated when co-culturing, the interaction outcomes may vary depending on the initial density [77]. Additionally, in environments with low carbon source concentrations, where growth was limited, competitive interactions may have been more pronounced than in environments with more abundant carbon sources [78].

Overall, although the current study has several limitations, our findings nevertheless shed light on the dual effects of carbon source diversity in complex, rich environments. Higher carbon source diversity is often considered to provide more niches for species to coexist in [56]. By contrast, greater diversity of carbon sources would also increase the overlap of resource niches, leading to competitive interactions between species. This latter effect may explain why competitive interactions were prevalent in complex environments containing diverse carbon sources [26–31].

## Supplementary Material

supplementary-information_wraf224

supplementary-tables_wraf224

## Data Availability

The datasets generated during this study are available on Zenodo [43]. The analysis codes are available on GitHub (https://github.com/HirokiOnoGit/Carbon-sources-shape-bacterial-relations). The sequence data are available in the DNA Data Bank of Japan (DDBJ) under the accession numbers LC893406–LC893413.
